# Functional characterization of genes encoding cadmium pumping P_1B_-type ATPases in *Aspergillus fumigatus* and *Aspergillus nidulans*

**DOI:** 10.1128/spectrum.00283-23

**Published:** 2023-09-07

**Authors:** Ildikó Vig, Zsigmond Benkő, Barnabás Cs. Gila, Zoltán Palczert, Ágnes Jakab, Fruzsina Nagy, Márton Miskei, Mi-Kyung Lee, Jae-Hyuk Yu, István Pócsi, Tamás Emri

**Affiliations:** 1 Department of Molecular Biotechnology and Microbiology, Faculty of Sciences and Technology, University of Debrecen, Debrecen, Hungary; 2 ELRN-UD Fungal Stress Biology Research Group, Debrecen, Hungary; 3 Department of Medical Microbiology, Faculty of Medicine, University of Debrecen, Debrecen, Hungary; 4 Biological Resource Center, Korea Research Institute of Bioscience and Biotechnology, Jeongeup-si, South Korea; 5 Department of Bacteriology, University of Wisconsin-Madison, Madison, Wisconsin, USA; Universidade de Sao Paulo, Ribeirao Preto, Sao Paulo, Brazil

**Keywords:** *Aspergillus fumigatus*, *Aspergillus nidulans*, PcaA cadmium pump, CrpA copper pump, heavy metal homeostasis, P_1B_-type ATPase, virulence, zinc toxicity

## Abstract

Several P_1B_-type ATPases are important Cd^2+^/Cu^2+^ pumps in *Aspergillus* species, and they are tightly associated with the heavy metal stress tolerance of these ascomycetous fungi. To better understand the roles of the two P_1B_-type ATPases, *Aspergillus nidulans* CrpA Cd^2+^/Cu^2+^ pump (orthologue of the *Candida albicans Crp1* Cd^2+^/Cu^2+^ pump) and *Aspergillus fumigatus* PcaA Cd^2+^ pump (orthologue of the *Saccharomyces cerevisiae* Pca1 Cd^2+^ pump), we have generated individual mutants and characterized their heavy metal susceptibilities. The deletion of CrpA in *A. nidulans* has led to the increased sensitivity of the fungus to stresses induced by Zn^2+^, Fe^2+^, or the combination of oxidative-stress-inducing menadione sodium bisulfite and Fe^3+^. Heterologous expression of *A. fumigatus* PcaA in the *S. cerevisiae pca1* deletion mutant has resulted in enhanced tolerance of the yeast to stresses elicited by Cd^2+^or Zn^2+^ but not by Fe^2+^/Fe^3+^ or Cu^2+^. Mammalian host immune defense can attack microbes by secreting Zn^2+^ or Cu^2+^, and the oxidative stress induced by host immune systems can also disturb metal (Cu^2+^, Fe^2+^, and Zn^2+^) homeostasis in microbes. In summary, PcaA and CrpA can protect fungal cells from these complex stresses that contribute to the virulence of the pathogenic *Aspergillus* species. Moreover, due to their presence on the fungal cell surface, these P_1B_-type ATPases may serve as a novel drug target in the future.

**IMPORTANCE:**

Mammalian host immune defense disrupts heavy metal homeostasis of fungal pathogens. P1B-type ATPase of *Aspergillus fumigatus* and *Aspergillus nidulans* may help to cope with this stress and serve as virulence traits. In our experiments, both *A. nidulans* Cd2+/Cu2+ pump CrpA and *A. fumigatus* Cd2+ pump PcaA protected fungal cells from toxic Zn2+, and CrpA also decreased Fe2+ susceptibility most likely indirectly. In addition, CrpA protected cells against the combined stress induced by the oxidative stressor menadione and Fe3+. Since P1B-type ATPases are present on the fungal cell surface, these proteins may serve as a novel drug target in the future.

## INTRODUCTION

*Aspergillus fumigatus* is one of the most prevalent filamentous fungal pathogens, causing life-threatening invasive aspergillosis in immunocompromised patients (1). Contribution of other *Aspergillus* species than *A. fumigatus* to fungal infections is also substantial: *Aspergillus nidulans*, known as a model organism in mycology, is responsible for the majority of invasive aspergillosis accompanied by chronic granulomatous disease (2 – 4). Due to the limitations of current therapies new strategies to increase therapeutic efficacy against aspergilli must be developed. P-type ATPases are considered as anti-fungal targets since they are easily accessible on the cell surface, and many play a pivotal role in the virulence of microorganisms (5 – 7).

P-type ATPases are a large and unique family of membrane proteins involved in various transport processes in nearly all cells. These ion pumps are quite widespread in eukaryotes, including fungi, and contribute to important physiological processes including the maintenance of ion homeostasis, membrane potential, and lipid-bilayer asymmetry, as well as the detoxification of transient metals (8). This latter phenomenon is attributed to P_1B_-type ATPases. These ATPases usually pump Ag^+^, Cu^+^, Cd^2+^, Co^2+^, Cu^2+^, Fe^2+^, Ni^2+^, Pb^2+^, or Zn^2+^ from the cell (8, 9). Some P_1B_-type ATPases are important virulence traits in bacteria and even in fungi (10 – 16). Their contribution to virulence is sometimes unclear. In the case of bacterial Fe^2+^-ATPases, it is assumed that Fe^2+^ efflux protects cells against iron overload, e.g., when bacteria escape from phagosomes to the relatively iron-rich cytosol (11). Alternatively, Fe^2+^-ATPases protect cells against toxic Fe^2+^ liberated within the cells under oxidative stress (11). Macrophages secrete Cu^2+^ and superoxide anion into the phagosomes to kill the embedded microbes, which may explain why microbial Cu^2+^ pumps like *A. fumigatus* CrpA or *Aspergillus flavus* CrpA and CrpB act as a virulence factor (13, 14).

Here, we have characterized the function of two P_1B_-type Cd^2+^ ATPases: CrpA (17, 18) of *A. nidulans* as an emerging opportunistic fungal pathogen (3) and PcaA (15, 19) of the well-known opportunistic human pathogen *A. fumigatus*. We show that the function of these Cd^2+^ pumps goes beyond protecting cells from this toxic heavy metal. They transport ions other than Cd^2+^ (e.g., Zn^2+^) and, due to the tight coupling between the metabolism of different metal ions, they may even affect the homeostasis of ions (e.g., Fe^2+^/Fe^3+^) that they are unlikely to transport. These properties of P_1B_-type microbial ATPases may explain why they have been identified as a virulence trait in many microorganisms (10 – 16). In the case of CrpA, we studied gene deletion strains to characterize the function of this protein. Recently, Bakti et al. (15) demonstrated that deletion of *pcaA* reduced the virulence of *A. fumigatus* in the *Galleria mellonella* infection model; however, the gene deletion mutant showed only increased Cd^2+^ but not Cu^2+^, Fe^2+^, or Zn^2+^ sensitivity (15). We speculated that, when *A. fumigatus* mutants were tested, the consequences of deletion/overexpression of the *pcaA* gene might have been masked or counteracted by elements of the heavy metal detoxification system other than PcaA. Therefore, we expressed *A. fumigatus pcaA* in *Saccharomyces cerevisiae* to study its functions in a host cell different from *A. fumigatus*. Understanding the contribution of P_1B_-type ATPases to metal homeostasis can promote research on these pumps as antifungal target.

## RESULTS AND DISCUSSION

The function of the fungal P_1B_-type ATPases has been extensively studied in *S. cerevisiae* and *Candida albicans. S. cerevisiae* has a copper (Ccc2) and cadmium (Pca1) P_1B_-type ATPase. Ccc2 belongs to the 1B-1 subfamily (9); it localizes in the trans-Golgi membrane and provides Cu^2+^ to the multicopper ferroxidase Fet3, thus indirectly participating in iron uptake (20). Pca1 is a member of the 1B-2 subfamily (9). In addition to the Cd^2+^ detoxification, Pca1 also contributes to the Cu^2+^ tolerance by sequestering Cu^2+^ in its Cys-rich N-terminal region and may also play a role in iron homeostasis (21, 22). *C. albicans* has two copper P_1B_-type ATPases, *Crp1* (Crd1) and Ccc2 (23 – 25). *Crp1* functions as a Cu^2+^, Cd^2+^, and Ag^+^ pump (23, 24). *C. albicans* Ccc2, like its *S. cerevisiae* orthologue, localizes in the Golgi membrane and indirectly affects iron uptake (25).

**Fig 1:**
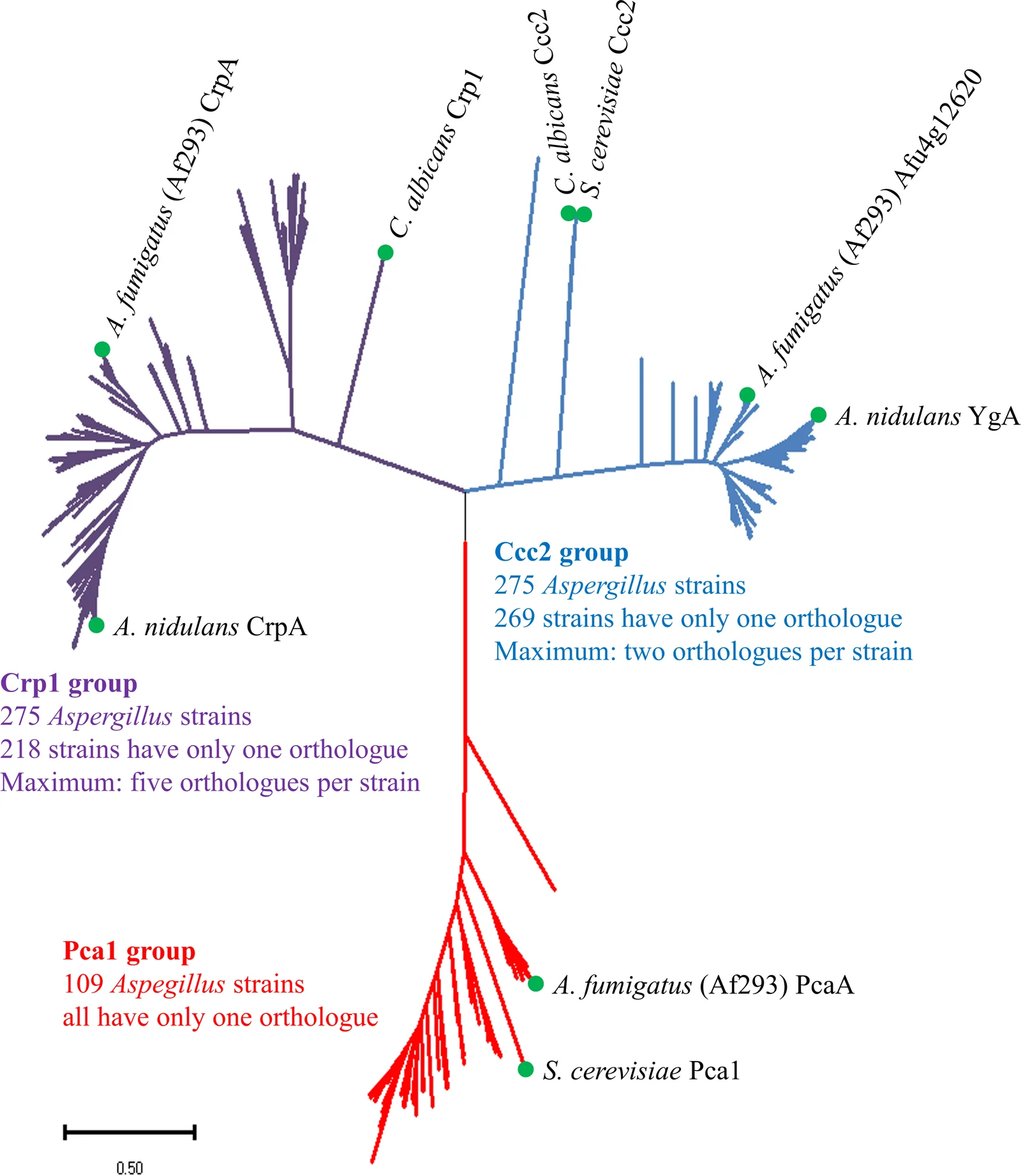
Phylogenetic analysis of the *Aspergillus* P_1B_-type ATPases.The evolutionary history of proteins was inferred using the maximum likelihood method and JTT matrix-based model conducted in MEGA11. For details, see Table S1.

The *Aspergillus* genomes studied (275 strains of 256 species) encode two to four, even up to eight, P_1B_-type ATPase genes (Table S1; Fig. 1). Each strain has at least one (maximum five) *Crp1* (CrpA) and one (maximum two) Ccc2 (CtpA) orthologues (Table S1; Fig. 1). Interestingly, the Pca1 orthologue PcaA is present in only 109 strains (Table S1; Fig. 1) (26). *A. nidulans* has two P_1B_-type ATPases, CrpA (orthologue of *C. albicans Crp1*) and YgA (orthologue of *C. albicans* Ccc2) (Table S1; Fig. 1). CrpA is responsible for Cu^2+^ and Cd^2+^ tolerance and can pump Ag^+^ (17, 18). YgA is involved in copper compartmentalization and provides Cu^2+^ for conidial pigmentation for the activity of the developmental phenol oxidase, IvoB (27). The genome of *A. fumigatus* (Af293) encodes three P_1B_-type ATPases (Table S1; Fig. 1). The *C. albicans Crp1* orthologue CrpA functions as a Cu^2+^ and Zn^2+^ pump (13, 28). The Afu4g12620 gene encodes a putative copper-transporting ATPase (29), which is the orthologue of *C. albicans* Ccc2 (Fig. 1; Table S1). PcaA (orthologue of *S. cerevisiae* Pca1) is involved in Cd^2+^ detoxification (15). PcaA was not revealed to be essential for wild-type-like Cu^2+^, Fe^2+^, or Zn^2+^ tolerance, but deletion of *pcaA* decreased, while overexpression of it increased, oxidative stress tolerance elicited by menadione sodium bisulfite (MSB) (15). The *A. fumigatus* Af293 strain, where *pcaA* is highly active, showed significantly stronger virulence in the mouse infection system than other wild-type strains with small or negligible *pcaA* activity (19). The deletion of *pcaA* also attenuated the virulence of *A. fumigatus* in the *G. mellonella* infection model. This phenotype was explained by the altered oxidative stress tolerance of the gene-deletion mutant, and this lack of PcaA may influence the activity of other metal homeostasis proteins involved in virulence (15). Note that the genome of *A. fumigatus* A1163 contains only *C. albicans Crp1* and Ccc2 orthologues but no *S. cerevisiae* Pca1 orthologue (Table S1). In fact, Barber et al. (30) found that unlike *crp1* and *ccc2*, which are part of the core genome of *A. fumigatus*, *pcaA* is an accessory gene occurring in only 2.67% of the studied 300 isolates. The data available at FungiDB (https://fungidb.org) also support this observation: Out of 879 whole genome sequenced isolates where the sample type (clinical vs environment) was clearly identifiable, only 32 (3.64%) isolates possessed the *pcaA* gene (Table S2). Importantly, 30 of the isolates having *pcaA* were clinical isolates, which represents a significant enrichment of the *pcaA* harboring isolates within clinical isolates (Fisher exact test; *P* = 0.0001767; Table S2). This enrichment suggests that although PcaA is not essential for virulence, possessing this gene can be beneficial for clinical isolates.

In order to better understand how PcaA contributes to the oxidative stress tolerance and virulence of *A. fumigatus* Af293, we expressed *pcaA* in *S. cerevisiae* and examined the phenotype of the mutant. It was hoped that this approach would reveal properties of PcaA that had not previously been found using *pcaA* gene deletion/overexpression strains. Moreover, to gain a broader understanding of the function of Cd^2+^-transporting P_1B_-type ATPases, we also investigated the function of *A. nidulans* CrpA, another known cadmium pump of aspergilli.

**Fig 2:**
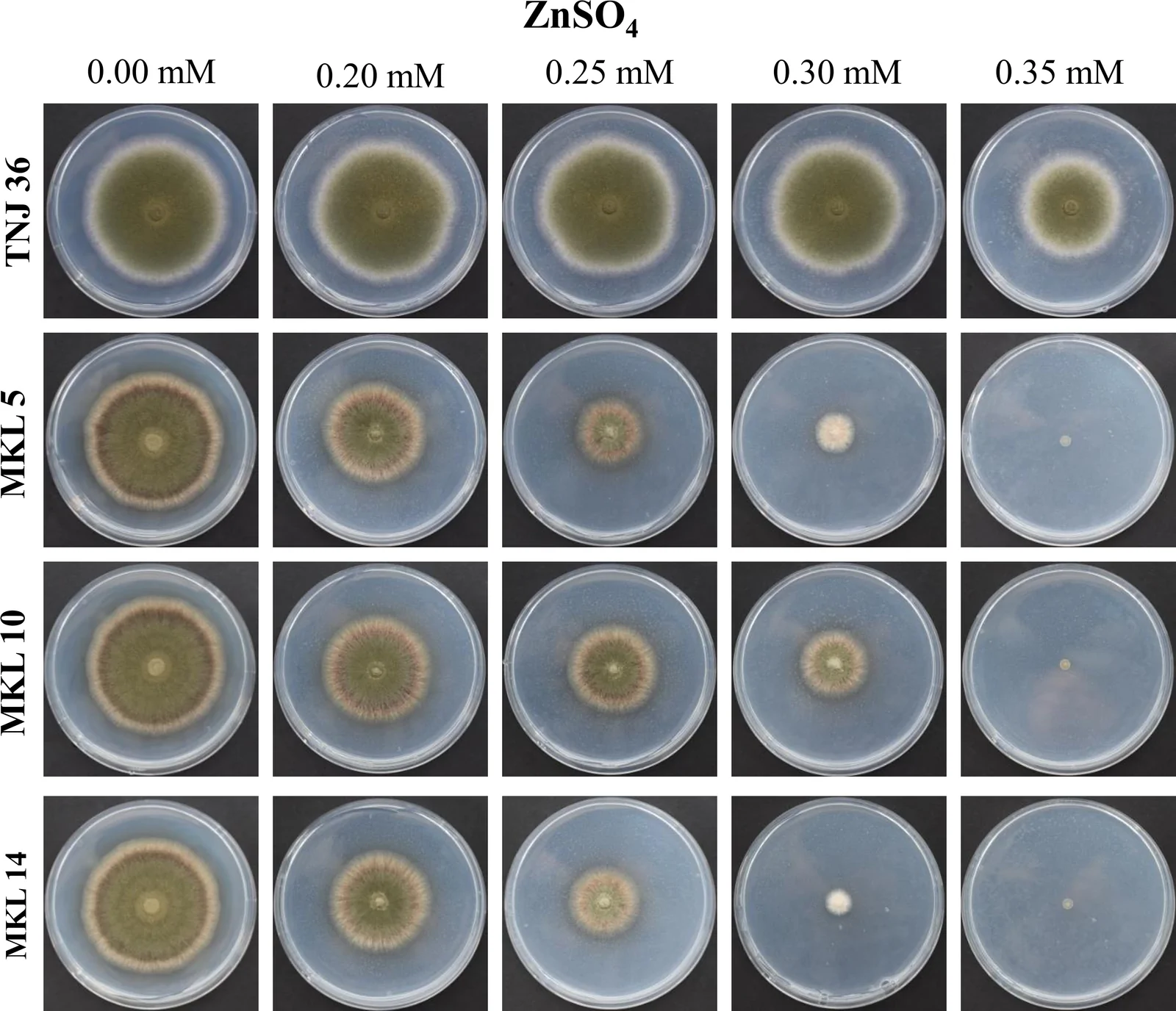
Comparison of ZnSO_4_ tolerance of the *ΔcrpA* ATPase mutants (MKL5, MKL10, and MKL14) and the reference (TNJ36) *A. nidulans* strains. Representative photos taken on the fifth day are presented. The Petri dish diameter is 85 mm.

**Fig 3:**
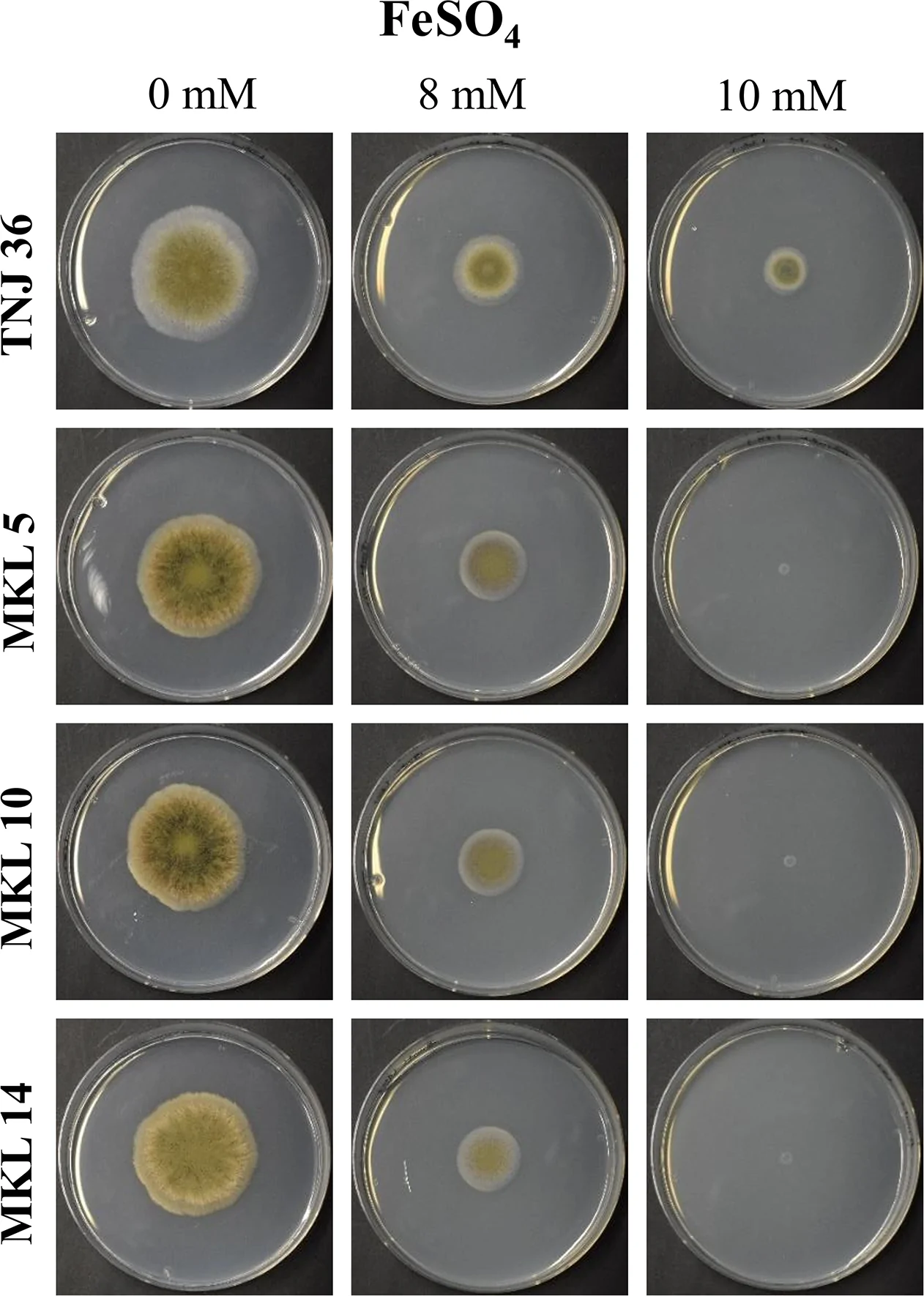
Comparison of FeSO_4_ tolerance of the *ΔcrpA* ATPase mutants (MKL5, MKL10, and MKL14) and the reference (TNJ36) *A. nidulans* strains. Representative photos taken on the third day are presented. The Petri dish diameter is 85 mm.

**Fig 4:**
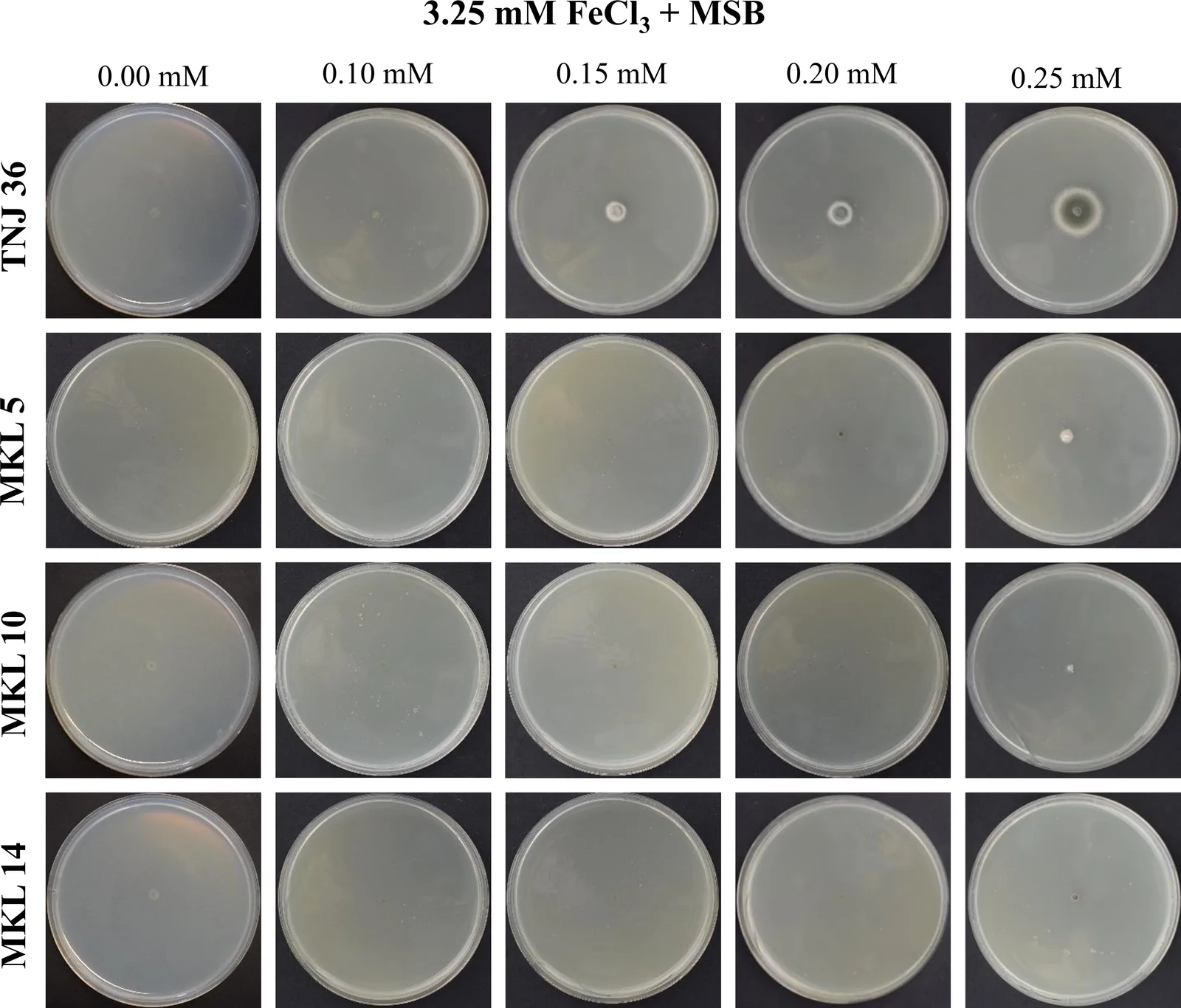
Comparison of MSB tolerance of the *ΔcrpA* ATPase mutants (MKL5, MKL10, and MKL14) and the reference (TNJ36) *A. nidulans* strains in the presence of 3.25 mM FeCl_3_. Representative photos taken on the fifth day are presented. The Petri dish diameter is 85 mm.

Regarding CrpA, we found that all the tested *A. nidulans ΔcrpA* strains were more sensitive to ZnSO_4_ treatment than the reference strain (Fig. 2; Table 1), suggesting the involvement of this pump in Zn^2+^ efflux, as was also found with *A. fumigatus* CrpA (28). The gene deletion strains showed increased Fe^2+^ sensitivity as well (Fig. 3; Table 1). In contrast, Fe^3+^ tolerance from the studied four strains and their MSB-elicited oxidative stress tolerance did not differ substantially from one another (Table 2; Fig. S1A and S1B). Interestingly, MSB and Fe^3+^ stresses showed an antagonistic effect when they were combined (Table 2; Fig. S1C): FeCl_3_ at 3 mM concentration completely inhibited the growth of the fungus. However, cultures were able to grow when MSB and 3 mM FeCl_3_ were added together (Table 2; Fig. S1C). MSB increases superoxide production in cells; superoxide reduces Fe^3+^ to Fe^2+^ and also destroys Fe-S cluster proteins (31). The buffered superoxide production due to the high Fe^3+^ concentration as well as the reduced Fe^3+^ levels due to Fe^3+^ - Fe^2+^ reduction and increased iron utilization may explain the observed antagonistic effect. When FeCl_3_ was applied at 3.25 mM concentration, a clear difference was found between the mutants and the reference strain: All the gene deletion strains showed decreased tolerance to the combined MSB-FeCl_3_ stress compared to the reference strain (Table 2; Fig. 4). These data suggest that CrpA contributes to iron metabolism as well. CrpA may pump out the excess Fe^2+^ from the cells or more likely mediate iron metabolism indirectly. Oxidative stress (induced by MSB) can disturb metal ion homeostasis and may liberate potentially harmful Zn^2+^ (32) and Cu^2+^. CrpA, by pumping these ions out of the cells, protects the fungus from this stress. Emerging data show that there is a tight association between the metabolism of different transition metals in fungi, in addition to the copper dependence of iron uptake via the reductive iron assimilation pathway, which has been observed in several fungi (20, 25, 33). It has also been shown that an increase in intracellular copper levels can elevate the iron content in *A. fumigatus* cells (34). Iron availability also regulates zinc metabolism mediated by the transcription factor ZafA (35), and in line with this, iron starvation has been shown to upregulate the vacuolar zinc transporter ZrcA (important in removing excess zinc from cytosol) and downregulate the zinc importer ZrpB in *A. fumigatus* (36). In addition, ZafA upregulates CtrC and CtrA2 copper transporters at low zinc concentrations (37). Increased extracellular Fe^2+^ or Fe^3+^ levels may disrupt the fine-tuned coordination of zinc-iron and/or copper-iron metabolism, resulting in the need for CrpA-mediated Zn^2+^ and/or Cu^2+^ efflux.

**TABLE 1:** Effect of ZnSO_4_ or FeSO_4_ on the growth of *ΔcrpA* (MKL 5, MKL 10, and MKL 14) and reference (TNJ 36) *A. nidulans* strains^*d*^

Additives	*A. nidulans* colony diameter (mm)^*a*^			
	TNJ 36	MKL 5	MKL 10	MKL 14
No ZnSO_4_ added	64.7 ± 0.6	60.3 ± 0.6^*a*^	59.7 ± 0.6^*b*^	59.3 ± 0.6^*b*^
2.0 mM ZnSO_4_	64.7 ± 0.6	43.3 ± 2.1^*b* *,* *c* *,* *d*^	50.3 ± 5.0^*b,c,d*^	44.0 ± 4.0^*b,c,d*^
2.5 mM ZnSO_4_	65.7 ± 0.6	29.0 ± 1.0^*b* *,c* *,^d^*^	39.3 ± 0.6^*b,c,d*^	35.3 ± 1.5^*b,c,d*^
3.0 mM ZnSO_4_	63.0 ± 1.0	17.0 ± 1.0^*b,c,d*^	27.0 ± 1.7^*b,c,d*^	11.7 ± 0.6^*b,c,d*^
3.5 mM ZnSO_4_	52.0 ± 2.0^*c*^	5.0 ± 0.0^*b,c,d*^	6.0 ± 1.7^*b,c,d*^	5.3 ± 0.6^*b,c,d*^
No FeSO_4_ added	45.7 ± 0.6	43.3 ± 1.2^*b*^	43.7 ± 0.6^*b*^	43.0 ± 1.0^b^
8.0 mM FeSO_4_	25.7 ± 0.6^*c*^	23.7 ± 0.6^*b* *,c*^	23.3 ± 0.6^*b,* *c*^	23.3 ± 0.6^*b* *,* *c*^
10 mM FeSO_4_	15.3 ± 0.6^*c*^	0^*b* *,c* *,* *d*^	0^*b* *,* *c* *,* *d*^	0^*b* *,* *c* *,* *d*^

^a^ Colony diameters of 5- (ZnSO_4_ treatment) or 3- (FeSO_4_ treatment) day-old cultures (mean ± SD; *n* = 3) are presented. Data were statistically analyzed with a two-way ANOVA followed by Tukey post-hoc test (*P* < 0.05).

^b^ Significant difference between the mutant and the reference strain.

^c^ Significant difference between the untreated and the appropriate stress-treated cultures.

^d^ Significant interaction between the effect of treatment (treated vs untreated) and gene deletion (mutant vs reference strain) in one (ZnSO_4_ or FeSO_4_ treatment) experiment.

**TABLE 2:** Effect of FeCl_3_, MSB, and combined FeCl_3_ and MSB treatments on the growth of *ΔcrpA* (MKL 5, MKL 10, and MKL 14) and reference (TNJ 36) *A. nidulans* strains^*d*^

Additives	*A. nidulans* colony diameter (mm)^*a*^			
	TNJ 36	MKL 5	MKL 10	MKL 14
No MSB added	64.3 ± 0.6	59.3 ± 1.2^*b*^	58.3 ± 0.6^*b*^	58.7 ± 0.6^*b*^
0.05 mM MSB	50.3 ± 0.6^*c*^	47.0 ± 1.0^*b,c*^	43.3 ± 0.6^*b,c*^	45.0 ± 1.0^*b,c*^
0.10 mM MSB	40.7 ± 0.6^*c*^	39.3 ± 0.6^*c,d*^	38.0 ± 2.6^*c*^	39.7 ± 1.5^*c,d*^
0.20 mM MSB	33.3 ± 1.5^*c*^	0.0 ± 0.0^*b,c,d*^	29.0 ± 2.0^*c*^	27.5 ± 7.5^*c*^
0.30 mM MSB	0.0 ± 0.0^*c*^	0.0 ± 0.0^*c*^	0.0 ± 0.0^*c*^	0.0 ± 0.0^*c*^
				
No FeCl_3_ added	62.3 ± 0.6	58.7 ± 0.6^*b*^	58.3 ± 0.6^*b*^	58.7 ± 0.6^*b*^
2.80 mM FeCl_3_	38.3 ± 0.6^*c*^	33.3 ± 2.1^*b,c*^	30.3 ± 5.0^*c*^	35.0 ± 5.2^*c*^
2.90 mM FeCl_3_	11.3 ± 4.0^*c*^	0.0 ± 0.0^b,c,d^	11.0 ± 4.2^*c*^	10.3 ± 2.1^*c*^
3.00 mM FeCl_3_	0.0 ± 0.0^*c*^	0.0 ± 0.0^*c*^	0.0 ± 0.0^*c*^	0.0 ± 0.0^*c*^
3.25 mM FeCl_3_	0.0 ± 0.0^*c*^	0.0 ± 0.0^*c*^	0.0 ± 0.0^*c*^	0.0 ± 0.0^*c*^
				
3 mM FeCl_3_	0.0 ± 0.0^*c*^	0.0 ± 0.0^*c*^	0.0 ± 0.0^*c*^	0.0 ± 0.0^*c*^
+0.10 mM MSB	31.7 ± 0.6^*c*^	30.3 ± 9.0^*c*^	32.0 ± 1.7^*c*^	31.0 ± 2.6^*c*^
+0.15 mM MSB	51.7 ± 1.5^*c*^	52.0 ± 1.0^*c*^	52.3 ± 1.2^*c*^	53.7 ± 2.1^*c*^
+0.20 mM MSB	47.7 ± 2.3^*c*^	49.0 ± 1.0^*c*^	48.0 ± 1.0^*c*^	47.3 ± 2.1^*c*^
+0.25 mM MSB	55.3 ± 3.2^*c*^	54.3 ± 1.5^*c*^	53.7 ± 1.5^*c*^	53.0 ± 1.0^*c*^
				
3.25 mM FeCl_3_	0.0 ± 0.0^*c*^	0.0 ± 0.0^*c*^	0.0 ± 0.0^*c*^	0.0 ± 0.0^*c*^
+0.10 mM MSB	0.0 ± 0.0^*c*^	0.0 ± 0.0^*c*^	0.0 ± 0.0^*c*^	0.0 ± 0.0^*c*^
+0.15 mM MSB	10.7 ± 1.2^*c*^	0.0 ± 0.0^*b,d*^	0.0 ± 0.0^*b,d*^	0.0 ± 0.0^*b,d*^
+0.20 mM MSB	11.3 ± 3.1^*c*^	2.7 ± 0.6^*b,c,d*^	0.0 ± 0.0^*b,c,d*^	0.0 ± 0.0^*b,c,d*^
+0.25 mM MSB	23.0 ± 1.0^*c*^	4.3 ± 1.5^*b,c,d*^	3.7 ± 0.6^*b,c,d*^	3.3 ± 0.6^*b,c,d*^

^a^ Colony diameters of 5-day-old cultures (mean ± SD; *n* = 3) are presented. Data were statistically analyzed with a two-way ANOVA followed by Tukey post-hoc test (*P* < 0.05).

^b^ Significant difference between the mutant and the reference strain.

^c^ Significant difference between the untreated and stress-treated cultures.

^d^ Significant interaction between the effect of treatment (treated vs untreated) and gene deletion (mutant vs reference strain).

**Fig 5:**
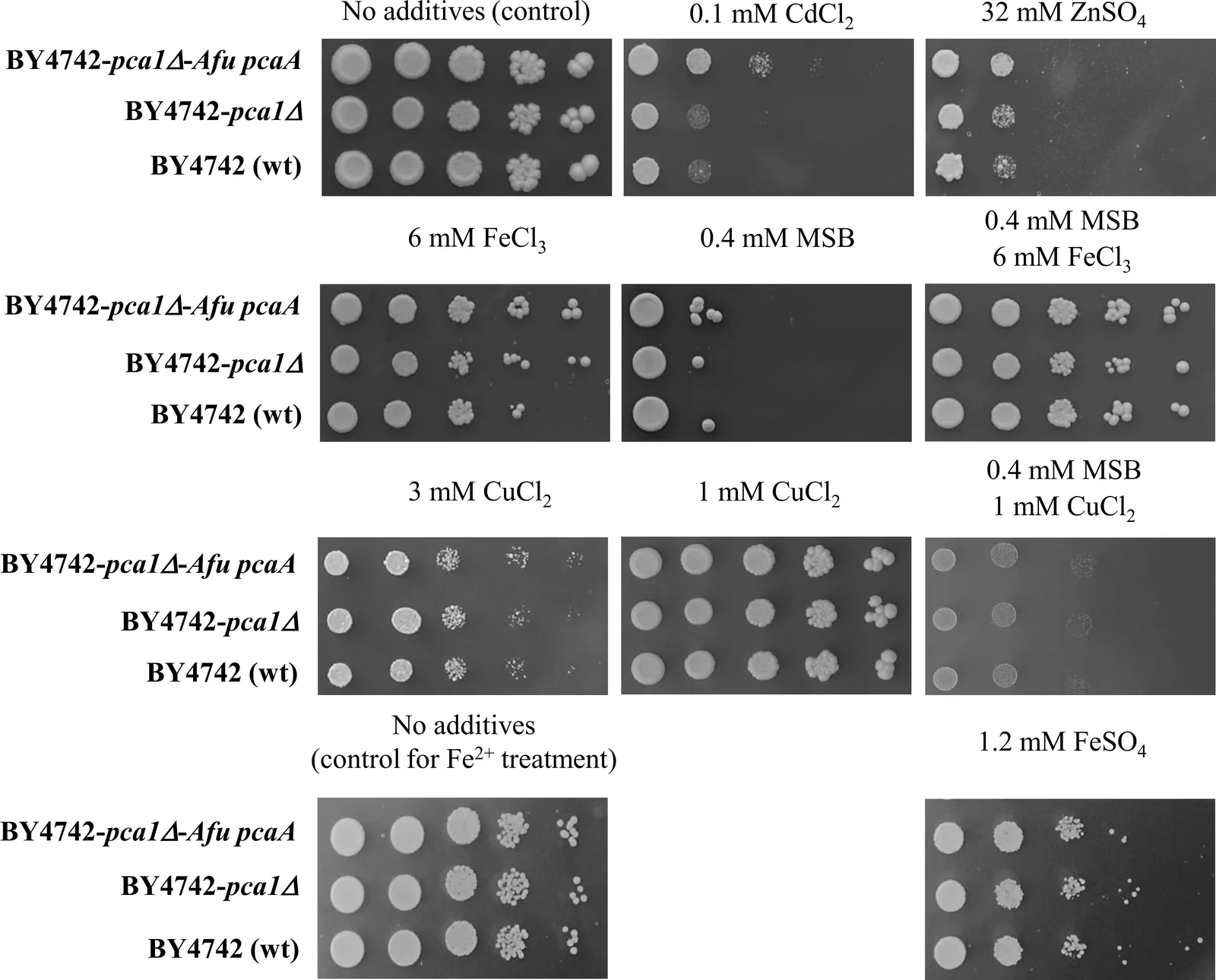
Stress tolerance attributes of *S. cerevisiae* (*pca1Δ*) expressing *A. fumigatus pcaA*. The starting OD_600_ was 0.4 (1×); 10×, 100×, 1,000×, and 10,000× dilutions were applied. Photos were taken on the second day.

**Fig 6:**
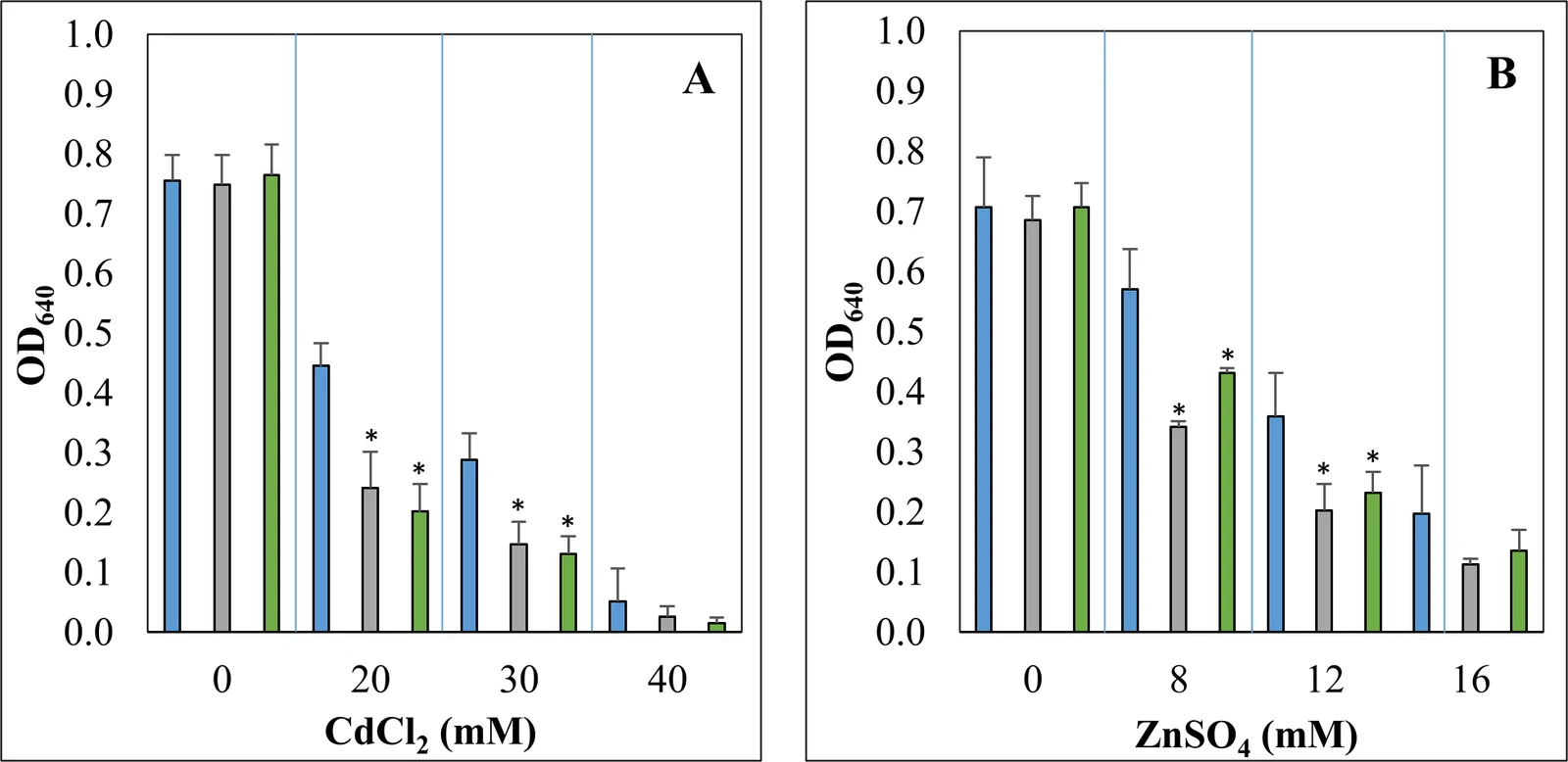
Effect of *A. fumigatus pcaA* expression on the of CdCl_2_ (**A**) and ZnSO_4_ (**B**) susceptibility of *S. cerevisiae*. The growth of three *S*. *cerevisiae* strains, the BY4742-*pca1ΔAfu pcaA* (an *A. fumigatus pcaA* expressing strain; blue), BY4742-*pca1*Δ (a *pca1* gene deletion mutant; grey), and BY4742 (a wild type laboratory strain; green) was studied using the broth microdilution method. Mean ± SD from four replicates are presented. * - Significant difference relative to *A. fumigatus pcaA*-expressing mutant at the studied concentration (Student’s *t*-test ; *P* < 0.05).

Expression of the *Afu-pcaA* gene in *S. cerevisiae* increased its CdCl_2_ tolerance relative to the *Sc-pca1* null mutant, as was expected (Fig. 5 and 6). No differences between the *S. cerevisiae pca1^+^* and *pca1^-^* strains were found, in line with the fact that Pca1 does not function as Cd^2+^ transporter in laboratory strains due to a missense mutation in its gene (22). The *Afu-pcaA* gene also increased ZnSO_4_ tolerance of the yeast (Fig. 5 and 6). The involvement of PcaA in zinc homeostasis was not recorded when a gene deletion strain was studied (15). It is possible that under the studied culturing conditions, some functions of PcaA were replaced by other zinc-exporting proteins like ZrcA (36) and CrpA (28). The involvement of PcaA in zinc homeostasis may explain how this cadmium pump contributes to the oxidative stress tolerance and virulence of *A. fumigatus* (15, 19): Mammalian hosts use Zn^2+^ to protect the mucosal surface against microbes (38), and oxidative stress can disrupt metal ion homeostasis, leading to the release of toxic Zn^2+^ within cells (39).

Regarding FeCl_3_, FeSO_4_, or MSB tolerance, no changes were recorded (Fig. 4). Interestingly, although *S. cerevisiae* Pca1 contributes to the Cu^2+^ detoxification by sequestering Cu^2+^ at its Cys-rich N-terminus region (21) and the Cys-rich N-terminus also occurs in *A. fumigatus* PcaA, expression of the *pcaA* gene in *S. cerevisiae* (Fig. 5) or the deletion of the *pcaA* gene in *A. fumigatus* (15) did not alter the Cu^2+^ susceptibility of the fungus. MSB and FeCl_3_ and MSB and CuCl_2_ have antagonistic effects on *S. cerevisiae* survival (Fig. 5). The observation that both iron and copper could suppress the growth inhibitory effect of MSB supports the hypothesis that these transition metals may act as redox buffers during MSB-induced oxidative stress.

Zinc and iron have been considered key players in host-pathogen interactions. Hosts sequester these essential metal ions from microbes as part of nutritional immunity (40). Not surprisingly, efficient iron and zinc ion acquisition mechanisms are essential for the *in vivo* virulence of pathogens (40, 41). In addition, microbes must cope with metal toxicity during infection. Mammalian hosts release Zn^2+^ onto the mucosal surface, which is toxic to pathogens, especially in combination with Mn^2+^ (38) or iron (36) withdrawal. Macrophages secrete Cu^2+^ into the phagosomes to kill the ingested microbes (13, 14). Moreover, iron-limited conditions of phagosomes and superoxide anion secreted into the phagosomes enhance copper toxicity further (34). Microbes may also suffer from iron/zinc overload when they escape from phagosomes to the cytosol (11). Besides these effects, microbes frequently must cope with oxidative stress during infection. Oxidative stress disturbs metal (Cu^2+^, Fe^2+^, Mn^2+^, and Zn^2+^) homeostasis, leading to metal toxicity. P_1B_-type ATPases, by secreting different metal ions, can protect microbes from these stresses, which explains why these ATPases are important virulence traits (10 – 16, 19).

The *A. nidulans* CrpA and *A. fumigatus* PcaA cadmium pumps can protect cells from zinc toxicity (Fig. 2, 5 and 6; Table 1), and CrpA also contributes to the Cu^2+^ detoxification (17, 18). Both ATPases increase the oxidative stress tolerance of the fungus (Table 2; Fig. 3) (15) presumably by stabilizing the perturbed metal ion homeostasis. In addition, CrpA can also reduce iron toxicity (Fig. 3 and 4; Tables 1 and 2) probably indirectly through the secretion of Cu^2+^ and/or Zn^2+^. Thus, these cadmium pumps may protect cells from dangerous metal ions they can pump (which are not limited to Cd^2+^) and may even reduce the toxicity of those they cannot pump due to the tight coupling of the metabolism of different metal ions (33 – 36). These properties of the two ATPases can contribute to the virulence of both *A. nidulans* (3) and *A. fumigatus* (1) and therefore represent potential targets for antifungal therapy.

## MATERIALS AND METHODS

### Strains and culture conditions

Strains listed in Table 3 were used in this study. *Aspergillus* strains were maintained on Barratt’s minimal nitrate agar plates, supplemented with pyridoxine, at 37°C (42). Conidia, freshly isolated from 6-day-old cultures, were used in all experiments.

**TABLE 3:** Strains used in the study

Strain	Feature	Genotype	Reference
*Aspergillus nidulans* TNJ36	Reference strain	*pyrG89*, *AfpyrG^+^*, *pyroA4*, *veA^+^*	18
*A. nidulans* MKL5	*ΔcrpA* mutant	*pyrG89*, *ΔcrpA*::*AfupyrG^+^, pyroA4*, *veA^+^*	18
*A. nidulans* MKL10	*ΔcrpA* mutant	*pyrG89*, *ΔcrpA*::*AfupyrG^+^, pyroA4*, *veA^+^*	18
*A. nidulans* MKL14	*ΔcrpA* mutant	*pyrG89*, *ΔcrpA*::*AfupyrG^+^, pyroA4*, *veA^+^*	18
*A. fumigatus* Af293	Source of *pcaA*	Wild type	43
*Saccharomyces cerevisiae* BY4742	Reference strain	*MATα his3∆1 leu2∆0 lys2∆0 ura3∆0*	44
*S. cerevisiae* BY4742-*pca1Δ*	*pca1^-^* strain	MATα his3∆1 leu2∆0 lys2∆0 *pca1Δ*	This study
*S. cerevisiae* BY4742-*pca1Δ-Afu pcaA*	*Afu-pcaA* expressing strain	MATα his3∆1 leu2∆0 lys2∆0 *pca1Δ – ppca1*::*Afu-pcaA*	This study

*S. cerevisiae* strains were selected and maintained on SC-dropout agar plates without uracil (SC plates) (45). Cultures were incubated at 30°C for 2 days, and these 2-day-old cultures were used to study their stress tolerance.

### Construction of *S. cerevisiae pca1*Δ and *pca1*Δ:*Afu-pcaA* strains

For amplification of *A. fumigatus pcaA*, total RNA was isolated from *A. fumigatus* Af293 cultures as described by Kurucz et al. (19) and reverse transcribed with First Strand cDNA Synthesis Kit (Thermo Scientific; Waltham, MA, USA) following the manufacturer’s protocol. The *pcaA* cDNA was amplified with the primer pair listed in Table 4.

**TABLE 4:** Oligonucleotides used in the study

Primer/oligo	Sequence
Primers for *A. fumigatus pcaA* amplification:	
pca-F	aaaaaaATGGGAGACGACTATTGCGGCC
pca-R	aacggtgactcgagtCTAGATCTTCGACCAGCGCAG
gRNA primers:	
PCA1-G1-F	gactttTAGCTACAAAAATTACAGGG
PCA1-G1-R	aaacCCCTGTAATTTTTGTAGCTAaa
Repair DNA primers:	
*for S. cerevisiae pca1Δ*	
*pca1Δ*_CRISPR_F	gatattttcgagatgcttccaggatttatacaatgaaagagcccaaagctgctgataacgCACTAACTAACTAAGCGTCG
*pca1Δ*_CRISPR_R	tcaaaaaaaaaaaagaaaagaaaaaagaaaatctacaatcaaatagcagcagtacctggaCGACGCTTAGTTAGTTAGTG
*for S. cerevisiae pca1Δ:Afu-pcaA*	
*pca1Δ:Afu-pcaA*_CRISPR_F	gatattttcgagatgcttccaggatttatacaatgaaagagcccaaagctgctgataacgATGGGAGACGACTATTGCGG
*pca1Δ:Afu-pcaA*_CRISPR_R	tcaaaaaaaaaaaagaaaagaaaaaagaaaatctacaatcaaatagcagcagtacctggaCTAGATCTTCGACCAGCGCA
Checking primers *S. cerevisiae pca1Δ and pca1Δ:Afu-pcaA strains*:	
pca1_F	aaaaaaATGAAGCCGGAAAAACTCTTC
pca1_R	aacggtgactcgagtCTAAATCTTTGCATAACGCAG

Genetic modification of *S. cerevisiae* BY4742 was carried out using the MoClo Yeast Tool Kit CRISPR/Cas9 system (Addgene, Watertown, MA, USA). The CRISPR guides and repair DNA oligos were designed using the Benchling online software (http://www.benchling.com). The single plasmid that expresses both the Cas9 nuclease and guide RNA cassette required for targeting the desired *pca1* locus was developed according to Lee et al. (46). The DNA oligos used are listed in Table 4. Both repair DNA fragments contained the same 60-bp-long flanking regions homologous to the upstream and downstream parts of *S. cerevisiae pca1* ORF. The *pca1Δ* repair DNA fragment contained in-frame stop codons as insert. The insert of the *pca1Δ:Afu-pcaA* repair DNA fragment consisted of *A. fumigatus pcaA* cDNA ORF, keeping the original *S. cerevisiae pca1* promoter. Repair DNA fragments were created with PCR using Dream Taq polymerase (Thermo Scientific, Waltham, MA, USA). *S. cerevisiae* BY4742 was transformed using the lithium acetate method (46). The *pca1* knock-out and Afu-*pcaA-*expressing mutants were verified by PCR using primer pairs listed in Table 4.

### Stress susceptibility tests

In the case of *A. nidulans* strains, Barratt’s minimal nitrate plates containing pyridoxine and supplemented with 0–35 mM ZnSO_4_, 0–3.25 mM FeCl_3_, or 0–0.3 mM menadione sodium bisulfite were point-inoculated with 5 µL fresh conidia suspension (10^5^ conidia/mL) and were incubated at 37°C for 5 days. The diameter of the colonies was recorded and used to characterize stress susceptibility. In some cases, MSB (0–0.25 mM final concentration) was added to media containing either 3 mM or 3.25 mM FeCl_3_. To test FeSO_4_ susceptibility, conidia were harvested in Barratt’s minimal nitrate medium, and the suspensions (10^5^ conidia/mL) were incubated for 8 h at 37°C. Pre-incubated (germinated) conidia were point-inoculated onto freshly prepared agar plates containing 0, 8, or 10 mM FeSO_4_, and the survival of the strains was monitored after 3 days of incubation at 37°C. Importantly, Fe^2+^ can be quickly (within hours) oxidized to Fe^3+^ under aerobic conditions. This experimental setup allowed us to test the effect of Fe^2+^ on the freshly formed hyphae. All experiments were carried out with three biological replicates. Since genetically manipulated mutants may harbor unexpected mutations and/or the genetic manipulation may have unexpected consequences that may affect their growth and stress sensitivity, three independent *crpA* gene deletion strains were studied, and only their shared phenotypes were discussed.

In the case of *S. cerevisiae* strains, overnight cultures were grown in 5 mL aliquots of SC-dropout broth without uracil (SC broth) at 30°C and 220 rpm. Aliquots (10 mL) of SC broth inoculated with overnight cultures (starting OD_600_ = 0.1) were incubated at 30°C and 220 rpm until the OD_600_ reached 0.4 value (approximately 4 h). Then, cultures were diluted (1×, 10×, 100×, 1,000×, or 10,000×) with sterile water, and 5 µL from each dilution was point inoculated on SC plates, and the SC plates were supplemented with 0.1 mM CdCl_2_, 0.4 mM MSB, 1 mM and 3 mM CuCl_2_, 6 mM FeCl_3_, 32 mM ZnSO_4_, or 0.4 mM MSB plus 6 mM FeCl_3_. Cultures were incubated at 30°C for 5 days. In the case of Fe^2+^ tolerance tests, cultures, after reaching the OD_600_ = 0.4 value, were either supplemented or not with FeSO_4_ (1.2 M final concentration) and were incubated for 0.5 h at 30°C and 220 rpm before point inoculation on SC plates. All experiments were carried out with three biological replicates.

The CdCl_2_ and ZnSO_4_ susceptibilities of *S. cerevisiae* strains were also tested using a broth microdilution method in SC broth in line with the CLSI standard M27-A3 guideline (47). Tests were performed in 96-well microtiter plates at 30°C. Each well contained 200 µL medium and was inoculated with approximately 10^3^ cells. The final metal concentrations were 0.8, 1.2, and 1.6 mM in the case of ZnSO_4_ and 0.2, 0.3, and 0.4 µM in the case of CdCl_2_. Note that due to the small inoculum size, the tested metal concentrations had to reduce markedly relative to those applied in agar plate tests. All strains were tested on four independent plates.

### *In silico* analyses of P_1B_-type ATPase orthologues in *Aspergillus*

Putative P_1B_-type ATPase orthologues were collected from the JGI MycoCosme database (https://mycocosm.jgi.doe.gov/mycocosm/home) using the blastp algorithm with default settings and *S. cerevisiae* Ccc2 as query sequence. Only hits with more than 800-bit score value were involved in the analysis. In the cases of *A. nidulans* and *A. fumigatus* (both Af293 and A1163), there were no hits in the bit score range of 400–800. The evolutionary history of the collected *Aspergillus* proteins as well as *S. cerevisiae* Ccc2, Pca1, and *C. albicans* Ccc2, *Crp1* proteins was inferred by the maximum likelihood method and JTT matrix-based model (48) using the MEGA11 software (49).

## SUPPLEMENTAL MATERIAL

Figure S1 spectrum.00283-23-s0001.pdf Susceptibility tests.

Table S1 spectrum.00283-23-s0002.xlsx List of *Aspergillus* P1B-type ATPases.

Table S2 spectrum.00283-23-s0003.xlsx Prevalence of *pcaA* possessing *A. fumigatus* isolates.

ASM does not own the copyrights to Supplemental Material that may be linked to, or accessed through, an article. The authors have granted ASM a non-exclusive, world-wide license to publish the Supplemental Material files. Please contact the corresponding author directly for reuse.
