# Supplementary material for: Functional characterization of genes encoding cadmium pumping P1B-type ATPases in Aspergillus fumigatus and Aspergillus nidulans
Source: Microbiol Spectr. 2023 Sep 7;11(5):e00283-23. doi: 10.1128/spectrum.00283-23 (PMC10581124; doi:10.1128/spectrum.00283-23)
Supplement: Figure S1 — Susceptibility tests. [file spectrum.00283-23-s0001.pdf]

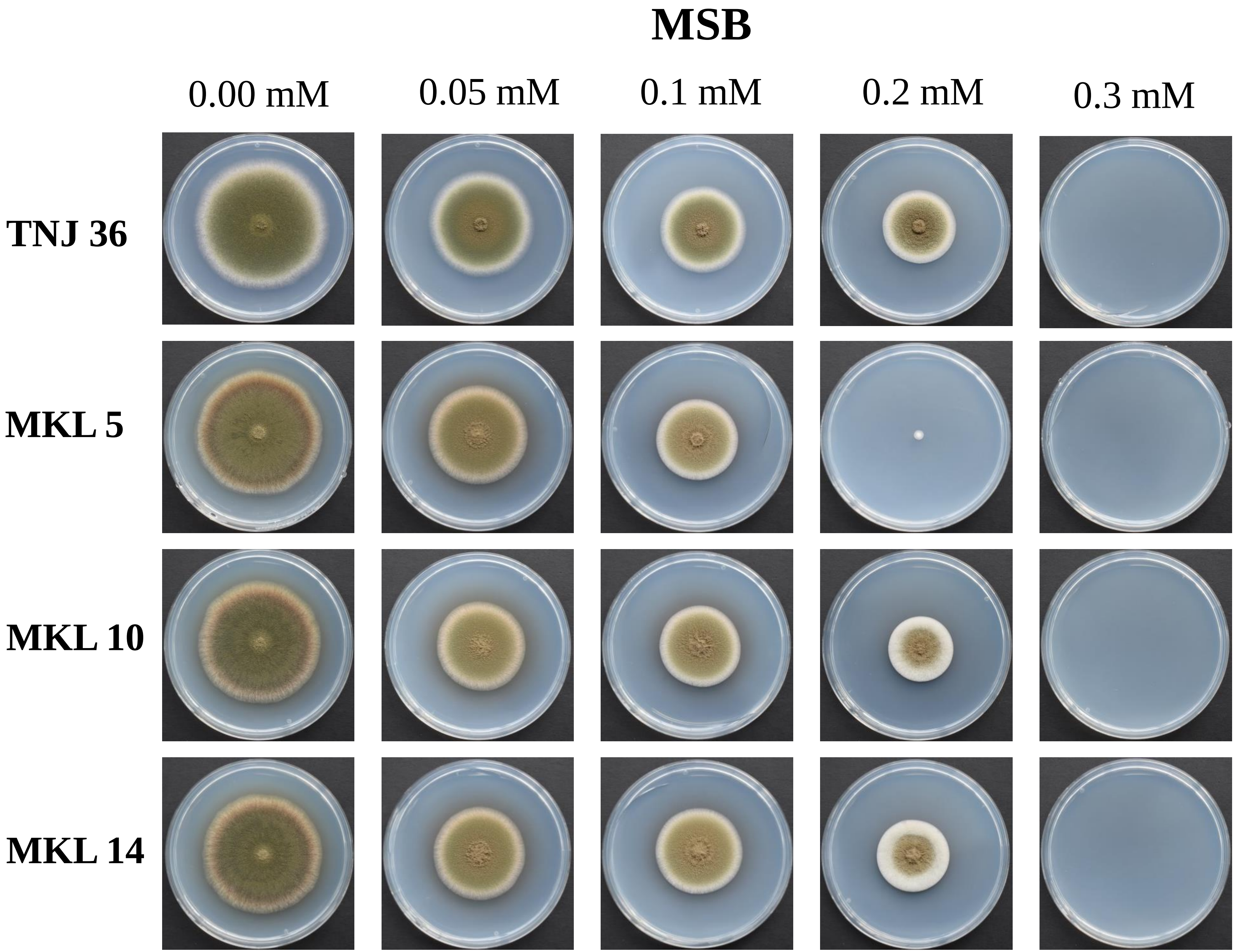

Fig. S1A Comparison of MSB tolerance of the *ΔcrpA* ATPase mutants (MKL5, MKL10, MKL14) and the reference (TNJ36) *A. nidulans* strains. Representative photos taken at the 5th day are presented. Petri dish diameter is 85 mm.

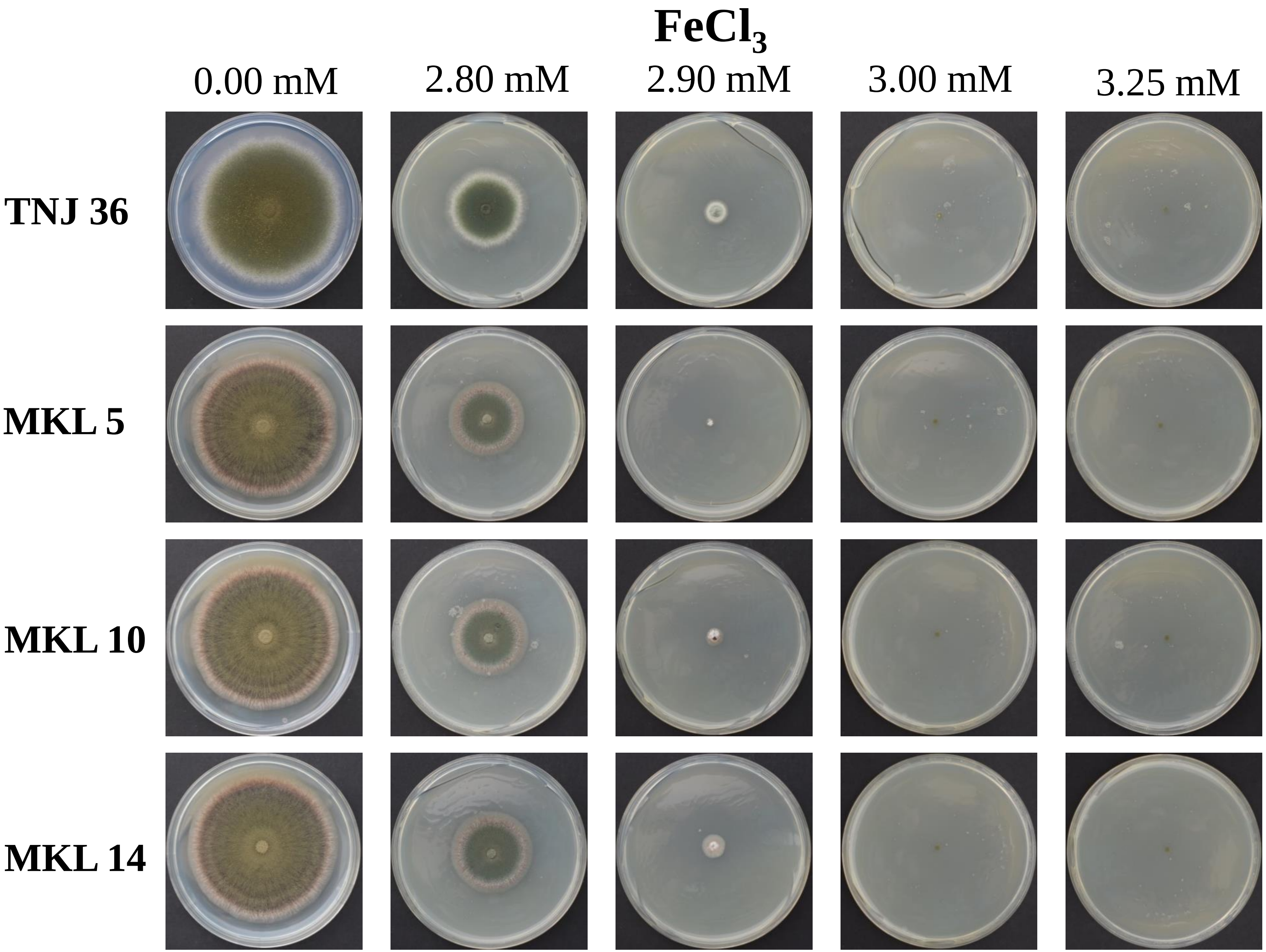

Fig. S1B Comparison of $FeCl_3$ tolerance of the *ΔcrpA* ATPase mutants (MKL5, MKL10, MKL14) and the reference (TNJ36) *A. nidulans* strains. Representative photos taken at the 5th day are presented. Petri dish diameter is 85 mm.

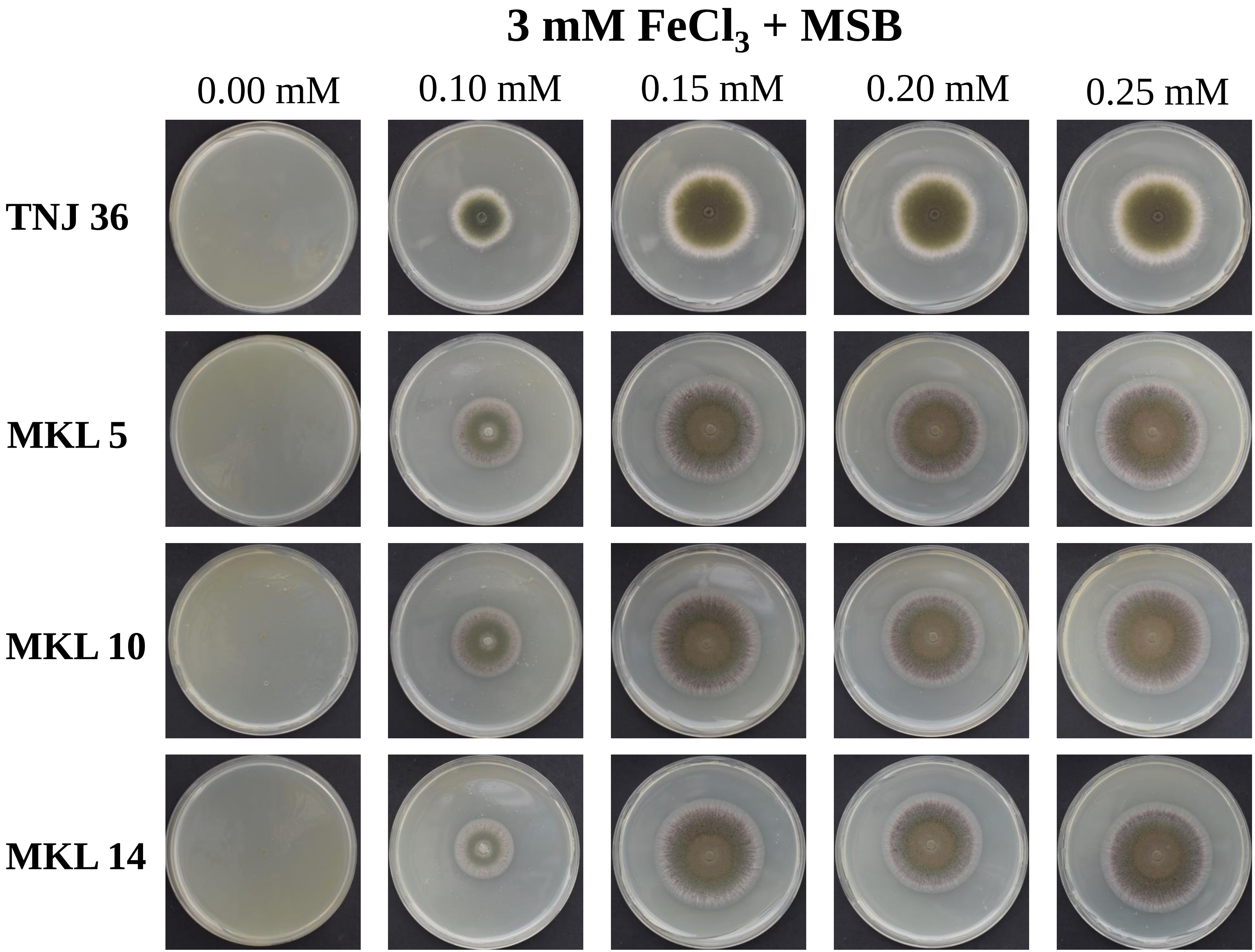

Fig. S1C Comparison of MSB tolerance of the *ΔcrpA* ATPase mutants (MKL5, MKL10, MKL14) and the reference (TNJ36) *A. nidulans* strains in the presence of 3 mM $FeCl_3$. Representative photos taken at the 5th day are presented. Petri dish diameter is 85 mm.
